# Synthesis and characterization of curcumin-loaded cellulose nanoparticles targeting bacterial quorum sensing and biofilms in foodborne bacteria

**DOI:** 10.3389/fmicb.2025.1680409

**Published:** 2026-01-21

**Authors:** Mohammad Zubair, Fohad Mabood Husain, Zahid Hameed Siddiqui, Altaf Khan Athar, Marai Alamri, Shoug Faisal Muhammad Ali Albudair

**Affiliations:** 1 Department of Medical Microbiology, Faculty of Medicine, University of Tabuk, Tabuk, Saudi Arabia; 2 Department of Food Science and Nutrition, College of Food and Agriculture Sciences, King Saud University, Riyadh, Saudi Arabia; 3 Department of Biology, Faculty of Science, University of Tabuk, Tabuk, Saudi Arabia; 4 Department of Pharmacology, College of Pharmacy, King Saud University, Riyadh, Saudi Arabia; 5 Department of Surgery, Faculty of Medicine, University of Tabuk, Tabuk, Saudi Arabia; 6 Faculty of Medicine, University of Tabuk, Tabuk, Saudi Arabia

**Keywords:** antimicrobial resistance, biofilm, curcumin loaded cellulose nanoparticles, food safety, quorum sensing

## Abstract

Over the past 25 years, antimicrobial resistance (AMR) has become a significant global health threat and a major cause of mortality. Foodborne diseases caused by drug-resistant bacteria capable of forming biofilms present serious health risks, necessitating innovative solutions for infectious disease management. Cellulose nanoparticles (CNPs), biocompatible and biodegradable, have found applications in targeted drug delivery, regenerative medicine, and tissue engineering. This study focuses on the synthesis and characterization of curcumin-loaded cellulose nanoparticles (CLCN) and their effects on quorum sensing (QS) and biofilm formation in both Gram-negative and Gram-positive foodborne bacteria (*Escherichia coli, Pseudomonas aeruginosa, Serratia marcescens*, *Chromobacterium violaceum*, and *Listeria monocytogenes*). FTIR confirmed molecular interactions between cellulose hydroxyl groups and curcumin. Thermal analysis (TGA/DSC) demonstrated enhanced structural stability with a gradual mass loss profile. Further, elemental composition analysis showed presence of carbon (50.6%) and oxygen (49.4%) in CLCN. CLCN exhibited MICs of 2 mg/mL against all test strains except in *L. monocytogenes* (8 mg/mL). At highest tested sub-MIC, violacein pigment was inhibited by over 58% in *C. violaceum* 12,472. CLCN disrupted pyocyanin, pyoverdin, LasB elastase, and rhamnolipid production by 53, 44, 39, and 47%, respectively. Exoprotease activity in test pathogens decreased by up to 58%. Biofilm production in all pathogens was significantly inhibited in the range of 48–68% at 0.5xMICs. Also, CLCN effectively removed preformed biofilms up to 46%. This study demonstrates that CLCN disrupt QS-regulated virulence traits and destabilizing biofilm architecture. By targeting virulence rather than growth, CLCN minimize the likelihood of resistance development and may serve as an adjunct or alternative to conventional antibiotic therapy. Thus, CLCN offer a biocompatible and sustainable antimicrobial strategy for food packaging systems, that limits surface-associated contamination and enhance food safety.

## 1 Introduction

Nanomaterials can be crafted from cellulose derived from diverse sources, including common vegetable cellulose found in wood (Gupta et al., 2018), bacterial cellulose, and plant cellulose (Long et al., 2018; Pircher et al., 2016; Revin et al., 2019). Bacterial cellulose is produced extracellularly, which helps them migrate toward oxygen-rich layers of the growth medium. Bacteria belonging to the genus *Komagataeibacter*, formerly *Gluconacetobacter*, emerge as the most promising bacterial cellulose producers (Yamada et al., 2012). Although it shows similarity with plant cellulose, bacterial cellulose exhibits structural distinctions. In pure form, its fibril thickness ranges from 20 to 80 nm, considerably less than plant cellulose, and it possesses a higher degree of crystallinity (Revin et al., 2018). Further, nanocellulose finds applications in biocompatible and biodegradable contexts, such as directed drug delivery in medicine and tissue engineering (Bhandari et al., 2017; Nita et al., 2020; Zheng et al., 2020). Their strong sorption capacity makes them effective for pollutant removal and insulation (Maleki, 2016; Nguyen et al., 2014; Wang et al., 2019). Cellulose-based hydrogels have also been developed for food packaging, functional foods, and food safety owing to their stimuli-responsive and structurally interchangeable properties (Nath et al., 2023).

A key limitation of cellulose is its inherently low reactivity, but this can be improved by functionalization or by incorporating organic antimicrobials, antibiotics, and other antimicrobial agents (Khattak et al., 2019; Klemm et al., 2005; Lemnaru et al., 2020). Several phytocompounds have also been utilized to improve the antimicrobial potential of cellulose (Isopencu et al., 2023). One such phytocompound, curcumin, a polyphenol found naturally in turmeric (*Curcuma longa*) has antimicrobial, antioxidant, anti-infective and anti-inflammatory properties (Zorofchian Moghadamtousi et al., 2014). Several reports have emerged where curcumin is crosslinked with collagen and chitosan/polyvinyl alcohol, and gelatin-based nanofiber membranes loaded with curcumin were synthesised (Abbas et al., 2019; Dharunya et al., 2016; Lv et al., 2022).

The growing danger of antimicrobial resistance (AMR) poses a significant risk to human health. Infections caused by pathogens resistant to multiple drugs are now among the top contributors to global death rates (Murray et al., 2022). Microbial exposure to higher antibiotic doses often accelerates the development of resistance (Ahmad et al., 2019; Witte, 2000). This growing resistance makes infections caused by foodborne pathogens an increasingly serious public health concern (Elbehiry et al., 2023). A major factor contributing to the persistence and virulence of foodborne pathogens is their ability to form biofilms, a complex, multicellular structure attached to surfaces and highly resistant to antimicrobial agents (Galié et al., 2018). *Staphylococcus aureus, Pseudomonas aeruginosa, Listeria monocytogenes, Serratia marcescens,* and *Escherichia coli* readily form biofilms in food-processing environments as well as during clinical infections. Once established within the human body, these biofilm-associated bacteria become difficult to eradicate, often resulting in persistent and recurrent infections (Liu et al., 2023). Biofilm development is closely regulated by quorum sensing (QS), a cell-density–dependent communication system. In this system, bacteria produce and respond to signaling molecules that coordinate gene expression, including genes responsible for biofilm formation and virulence (Miller and Bassler, 2001; Rutherford and Bassler, 2012). The majority of genes expressed during QS are secondary genes that are not essential for regular bacterial growth and division (Pena et al., 2019). The rise of AMR and slow antibiotic discovery have prompted interest in molecules that reduce pathogenicity rather than growth, thereby lowering the risk of resistance (Ahmad et al., 2019). Many investigations revealed that biodegradable nanocarriers such as chitosan, liposomes, and PLGA have shown QS attenuation and biofilm suppression (Nag et al., 2021; Padaga et al., 2024; Shariati et al., 2022). Previous studies involving curcumin-loaded biopolymers have primarily focused on antimicrobial activity against one or more pathogens (Anbari et al., 2022; Memar et al., 2025; Trigo-Gutierrez et al., 2021). In a recent investigation, multifunctional chitosan curcumin–tannic acid biocomposites have been shown to disrupt QS pathways and inhibit biofilm formation in pathogenic bacteria (Khan et al., 2024). However, studies employing biodegradable, cellulose-based nanocarriers using curcumin have not been well explored for their mechanistic effects on virulence regulation and biofilm inhibition/disruption.

This study was designed to manufacture curcumin-loaded cellulose nanoparticles (CLCN) and conduct a thorough characterization. The synthesized CLCN underwent testing to assess their impact on the modulation of QS-regulated virulence functions in foodborne pathogenic bacteria. Additionally, a comprehensive examination of the inhibition of biofilms against *E. coli*, *P. aeruginosa*, *S. marcescens*, *L. monocytogenes* and *C. violaceum* was performed.

## 2 Materials and methods

### 2.1 Synthesis of curcumin-loaded cellulose nanoparticles

#### 2.1.1 Cellulose nanocrystals

In this study, the method of Rubentheren et al. (2015) for preparing cellulose nanocrystals (CNCs) was modified slightly. In a beaker, 10 g of microcrystalline, dry powder of cellulose (Sigma-Aldrich, USA) were mixed with 3,000 L of distilled water (ratio 1:30) to begin the process. The mixture was kept under continuous stirring in an ice bath, and concentrated H_2_SO_4_ (95–98%, w/w) was added dropwise to achieve a final acid concentration of 64% (w/w), ensuring controlled hydrolysis. A 90-min stir at 45 °C was needed to prepare the suspension. The obtained suspension was centrifuged for 10 min at 6000 rpm, and cooled distilled water was added 1:10 ratio after acid hydrolysis. To remove excess acid, the suspension was neutralized by the dropwise addition of dilute NaOH solution (0.1 M) under continuous stirring until the pH reached 7.0. The neutralized material was then washed several times with deionized water and dialyzed for 48 h to remove residual sulfate ions. After neutralization, 20 min of ultrasonic treatment completed the dispersion. For bacterial growth prevention, a few drops of chloroform were added to the solution. Using a refrigerated in an airtight container, the filtered suspension was evaporated overnight to prepare dry CNCs.

#### 2.1.2 Curcumin-loaded cellulose nanoparticles

To prepare the CNC stock suspension, powdered CNC (2.5 gm) was dispersed in 250 mL of deionized water to prepare the CNC stock suspension. The suspension was stirred thoroughly and then sonicated using an ultrasonic bath for 10 min to ensure homogenous dispersion. The pH of the CNC suspension was adjusted using 1.0 M HCl to optimize interaction with curcumin. Separately, 50 mg of curcumin was dissolved in 5 mL of dichloromethane (DCM) to obtain a concentration of 10 mg/mL. This curcumin solution was then diluted with 20 mL of distilled water and stirred continuously for 24 h to facilitate proper dispersion.

The CNC suspension (1% w/v) was added slowly to the curcumin solution (10 mg/mL) under continuous stirring and allowed to mix for an additional 2 h to ensure effective loading of curcumin onto the cellulose nanocrystals. The resulting mixture was then cast onto a clean glass Petri dish, where the CLCN coagulated upon contact with deionized water. The formed nanoparticles were washed at least five times with distilled water to remove residual DCM and unbound curcumin. Finally, the purified cellulose/curcumin nanoparticles were dried in an oven at 60 °C for 12 h before being characterized for further studies.

### 2.2 Characterization of curcumin-loaded cellulose nanoparticles (CLCN)

#### 2.2.1 Fourier-transform infrared spectroscopy (FTIR) analysis

FTIR spectroscopic analysis was employed to investigate inter- and intra-chemical interactions among CLCN, utilizing the Thermo Nicolet 380 spectrometer (Thermo Electron Corporation, USA). The spectral data were obtained with 2 cm^−1^ intervals, spanning from 4,000 to 400 cm^−1^ in reflectance mode.

#### 2.2.2 Thermogravimetric analysis (TGA)

TGA was conducted to examine the thermal properties of CLCN, employing the TGA Q500 (TA instruments, USA). The samples underwent heating from 25 °C to 600 °C with increasing temperature by 5 °C per minute.

#### 2.2.3 Differential scanning colourimetry (DSC)

DSC was employed to assess the thermal stability of the synthesized CLCN using the DSC Q2000 differential scanning colourimeter (TA Instruments, USA). The sample was heated from 25 °C to 600 °C with increasing temperature by 5 °C per minute.

#### 2.2.4 Scanning electron microscopic (SEM) analysis

The network structure and morphology of CLCN were scrutinized utilizing the JEOL JSM 6510 LV (Tokyo, Japan). For this examination, 1–2 mg of the material was affixed to the stub and subjected to gold sputter coating for 2 to 3 min. Subsequently, sputtered samples were observed under electron beam to investigate the topography of their surface. The elemental composition analysis of CLCN was carried out using Oxford INCAx-sight EDAX, which is integrated with the SEM.

### 2.3 Growth conditions of bacterial cultures used

For this study, foodborne pathogens, i.e., *Pseudomonas aeruginosa* PAO1, *Escherichia coli* ATCC 25922, *Serratia marcescens* MTCC 97, and *Listeria monocytogenes* ATCC 19114 were used. All bacteria were grown in Luria-Bertani (LB) broth at their respective growth conditions. All assays of biofilms and quorum sensing were performed in LB broth unless stated.

### 2.4 Minimum inhibitory concentration (MIC) evaluation

The MIC of the CLCN against the test pathogens was determined using 2,3,5-triphenyltetrazolium chloride (TTC) dye, using a broth dilution assay as described earlier (Qais et al., 2018). Serial two-fold dilutions of the CLCN were prepared in Mueller–Hinton Broth (MHB) in a 96-well microtiter plate, with final concentrations ranging from 8 mg/mL to 0.0001 mg/mL. The bacterial cultures were adjusted to a final inoculum of approximately 5 × 10^5^ CFU/mL in each well. The plates were incubated at 37 °C for 18 h to allow bacterial growth. Group without the CLCN serving as the negative control. Azithromycin was used as a positive control for growth inhibition. Afterwards, ten microliters of TTC solution per well were added to detect the bacterial growth for 20 min. Pink or red colour in wells indicated the presence of bacterial cells that are metabolically active. The lowest dose of CLCN at which no red/pink colour was seen was considered as MIC.

### 2.5 Violacein pigment production

The extraction and quantitative assessment of violacein were conducted following a previously described method (Matz et al., 2004). To summarize, *C. violaceum* 12,472 was grown in LB medium overnight, both with and without CLCN. After the overnight incubation, 1 mL of the cultured bacteria was centrifuged at 10,000 rpm for 5 min to separate the violacein. The resulting pellet was then resuspended in DMSO (1 mL) and vortexed for 1 min to dissolve the violacein. This suspension was then centrifuged again for 5 min at 10,000 rpm to remove the bacterial debris/cells, and the optical density of the supernatant was taken at 585 nm. Violacein inhibition was expressed as a percentage relative to the untreated control.

### 2.6 Assay for prodigiosin production

For the assessment of prodigiosin production, *S. marcescens* MTCC 97 was cultivated both with and without CLCN for 24 h. After cultivation, cells were collected through centrifugation, and the supernatant was removed. Prodigiosin was extracted from the cells using 1 mL solution of acidified ethanol. The absorbance of samples was taken at 534 nm for quantification of the pigment (Slater et al., 2008).

### 2.7 Assessment of *P. aeruginosa* PAO1 virulence factors

The pyocyanin assay was executed in *Pseudomonas* broth (PB) medium, consisting of MgCl_2_ (1.4 g/L), peptone (20 g/L), and K_2_SO_4_ (10 g/L), which is known to enhance the pyocyanin, following the standard procedure (Husain et al., 2017). The bacteria, *P. aeruginosa* PAO1, was grown in PB medium with and without CLCN for 18 h. Following bacterial growth, chloroform (3 mL) was used to extract the pyocyanin from culture supernatant (5 mL). The organic phase was taken and re-extracted in HCl (1,200 μL). The optical density of the resulting red/pink aqueous phase was taken at 520 nm. Pyocyanin concentration is expressed in μg/ml, was determined by using earlier described calculations (Kurachi, 1958). The pyoverdin assay was conducted following the previously described method (Ankenbauer et al., 1985). In summary, overnight culture, both without and with CLCN, was centrifuged to get the supernatant. 100 μL of the supernatant was added to 900 μL of Tris–HCl (50 mM, pH 7.4). The fluorescence emission signal of the solution was taken at 460 nm with excitation at 400.

Elastolytic activity was assessed using elastin congo red (ECR) as described in a previous study (Kessler et al., 1982). Cell-free supernatant (100 μL) from both untreated and CLCN-treated *P. aeruginosa* PAO1 was mixed with 900 μL of ECR buffer (5 mg/mL ECR and 1 mM CaCl_2_ in 100 mM Tris, having pH 7.5) and placed in a shaking incubator for 3 h at 37 °C. 1 mL of sodium phosphate buffer was added to stop the reaction, and then placed on ice packs for half an hour. Using centrifugation, insoluble ECR was pelleted down, and absorbance was measured at 495 nm. The rhamnolipid assay was conducted using the orcinol method as previously described (Husain et al., 2017). 300 μL culture supernatant from both CLCN-treated and untreated cells was extracted in 600 μL of diethyl ether. The aqueous phase was discarded, and the organic phase was collected. The collected phase was evaporated to dry in 37 °C incubator. The sample was then reconstituted in 100 μL deionized water. 900 μL of orcinol solution (190 mL/mL orcinol in 53% H_2_S0_4_) was mixed with 100 μL sample. The resulting sample was heated for half an hour at 80 °C and then cooled for 15 min. The absorbance of samples was taken at 421 nm. For all virulence-factor assays, percent inhibition was calculated relative to the untreated control, which was considered 100% activity.

### 2.8 Assessment of exoprotease activity

The exoprotease activity of test bacteria was assessed through azocasein degradation method, following the standard protocol (Husain et al., 2017). In short, 100 μL of supernatant from untreated and CLCN-treated bacteria was combined with 1,000 μL azocasein (0.3%, pH 7.5), and then incubated for 15 min at 37 °C. To stop the reaction, 500 μL TCA was added and centrifuged for 12 min at 12000 rpm. The pellet was discarded, and the absorbance of the supernatant was taken at 400 nm.

### 2.9 Cell surface hydrophobicity (CSH)

The CSH was assessed using xylene, following a previously outlined procedure (Rosenberg et al., 1980). In brief, 1 mL of an overnight-grown culture of test bacteria was mixed with 0.1 mL of xylene and then different concentrations of CLCN were added. The control group consisted of xylene with cells, without CLCN. Vigorous vortexing of the cells was done for 2 min and then placed for 10 min for phase separation. Aqueous phase was taken, and its absorbance was monitored at 530 nm. % hydrophobicity was computed using the formula (1):


$$\%\text{hydrophobicity}=[1-\frac{\mathrm{OD}\;\text{after vortexing}}{\mathrm{OD}\;\text{before vortexing}}]\times 100\;\;$$ (1)

### 2.10 Measurement of exopolysaccharides

The quantification of exopolysaccharides (EPS) levels in both control and CLCN-treated cells was conducted, adhering to the standard procedure with minor changes (Hasan et al., 2019). In brief, test bacteria were cultivated with and without CLCN for 24 h. For the control group, no treatment was given. After incubation, cultures were subjected to centrifugation, and the supernatant was obtained. To precipitate EPS, prechilled ethanol was added to the supernatant and incubated at 4 °C overnight. The quantification of EPS was carried out by assessing the sugar content using the Dubois protocol (DuBois et al., 1956).

For all biofilm-associated virulence factors, percent inhibition was calculated using the untreated control as the reference (100% activity).

### 2.11 Inhibition of biofilm formation

Biofilm inhibition experiments were conducted using a 96-well microtiter plate, following previously outlined procedures (Husain et al., 2017). Overnight cultures of *E. coli* ATCC 25922, *P. aeruginosa* PAO1, *L. monocytogenes* ATCC 19114, and *S. marcescens* MTCC 97 were taken and diluted to approximately 10^6^ CFU/mL. A volume of 150 μL of this bacterial suspension was added to each well of a sterile 96-well microtiter plate. At 0.5xMIC, CLCN and curcumin were applied separately as the treatment groups. Azithromycin at respective sub-MICs was taken as positive control for biofilm inhibition. Control groups, lacking any treatment, were also included, and the plate was again placed in the incubator for 24 h. After incubation, the removal of planktonic cells and excess broth involved three washes in sterile buffer. The plate was then dried for 15 min. To stain the developed biofilms, 0.2 mL crystal violet solution was added and incubated for 20 min, followed by a gentle wash with sterile buffer, to eliminate the unbound crystal violet. The bound dye was dissolved in ethanol, and absorbance was measured at 620 nm. All treatments and controls were assayed in triplicate, and the experiment was repeated independently three times. Percent biofilm inhibition was calculated relative to the untreated control using the formula (2):


$$[\%\text{biofilm inhibition}=1-(\begin{array}{l} OD_{620}\;\text{treated}/\\ OD_{620}\;\text{control} \end{array})]\times 100]\;$$ (2)

Furthermore, in order to assess the impact of CLCN on the biofilm structures, confocal laser scanning microscopy (CLSM) was performed in the representative bacterial strains (*E. coli* ATCC 25922 and *P. aeruginosa* PAO1). Briefly, biofilms of test bacterial strains were developed on the cover slips as described above. After incubation, planktonic cells were removed, and cover slips were gently washed using sterile phosphate buffer. Biofilm cells were stained with 0.1% acridine orange for 15 min in the dark. After removing excess dye with gentle washing, the coverslips were air-dried and photographed using a confocal laser scanning microscope (Zeiss, Germany).

### 2.12 Eradication of formed biofilms

The assay for disrupting biofilms was conducted in 96-well plates, following a standard protocol (Qais et al., 2018). All test bacteria were cultivated in microtiter plates for 24 h to allow the formation of biofilms in plate wells. Subsequently, the media was gently removed, and sterile buffer was employed for washing to eliminate unattached bacterial cells. Fresh LB broth was introduced into the plate wells, followed by the addition of CLCN. Wells having bacterial culture without any treatment were taken as a negative control. The plate was again incubated for 24 h. Post-incubation, the media in plate wells were removed and washed with sterile buffer to eliminate unbound planktonic cells. The biofilm-attached cells were stained with crystal violet solution for 20 min. After staining, the excess stain was removed, and a gentle wash was performed. Finally, ethanol was used to dissolve biofilms, and absorbance was taken at 620 nm. The percentage of disruption was computed relative to the untreated control group.

### 2.13 Statistical analysis

All experiments were carried out in triplicate, and results are expressed as mean ± SD. Differences among treatment groups were analyzed using one-way ANOVA, with statistical significance set at *p* < 0.05. Significance levels in figures are indicated by *p* < 0.05 and *p* < 0.01.

## 3 Results and discussion

### 3.1 Fabrication of curcumin-loaded cellulose nanoparticles (CLCN)

CLCN synthesized using the methodology described in the previous section were stored at room temperature. The nanoparticles were characterized using certain microscopic and spectroscopic techniques.

### 3.2 Characterization

#### 3.2.1 Fourier-transform infrared spectroscopy

**Figure 1:**
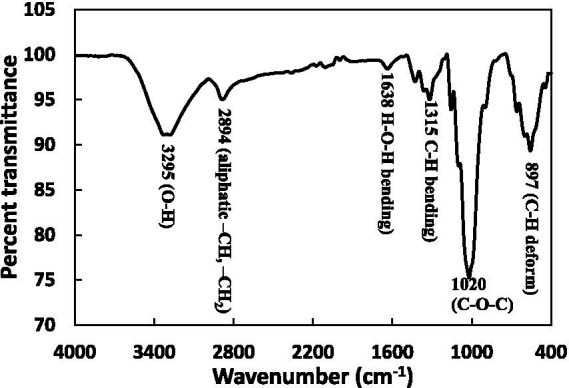
Fourier-transform infrared spectroscopy (FTIR) spectra of CLCN from 4,000 to 400 cm^−1^.

The cross-linking of cellulose plays a pivotal role in ensuring its conformational integrity and structural stability. Analysing the physicochemical changes in CLCN is crucial, as any alterations in structure can impact their biological properties. FTIR analysis was conducted to validate intermolecular interactions, as depicted in Figure 1, showcasing the FTIR spectra of CLCN. In the IR spectrum of CLCN, distinctive absorption peaks characteristic of the polymer was observed. Notably, the absorption peak within the 3,000 to 3,600 cm^−1^ which is due to stretching vibrations of hydroxyl groups of cellulose engaged in hydrogen bond formation (Revin et al., 2020). A less pronounced band in the 2,800 to 3,000 cm^−1^ range is attributed to stretching vibrations of the C–H bonds of methylene groups of cellulose (Kondo and Sawatari, 1996). A minor peak around 1,650 cm^−1^ is attributed to the deformation of –OH groups vibrations from adsorbed water. The 1,430 cm^−1^ peak is induced by symmetric –CH2 bending (Cael et al., 1975). Furthermore, the prominent band at 1020 cm^−1^ aligns with valence vibrations of C–O bonds (Revin et al., 2020). Overall, the FTIR spectra provide insights regarding the chemical bonds and vibrations of bonds present in CLCN.

#### 3.2.2 Thermogravimetric analysis (TGA)

**Figure 2:**
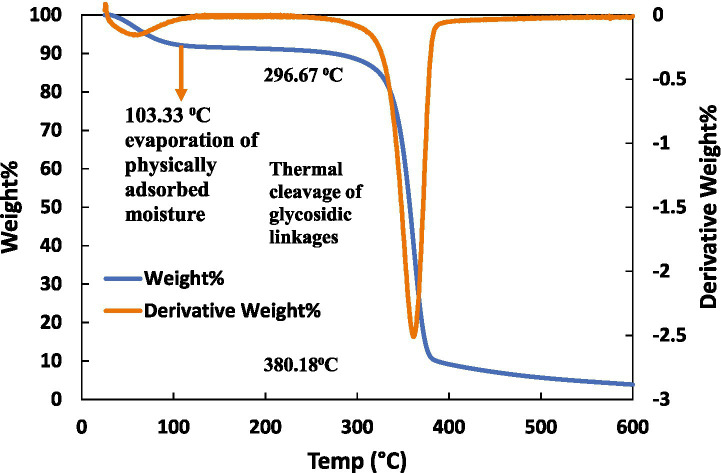
Thermogravimetric analysis (TGA) graphs of the CLCN. The initial weight loss till 100 °C came from the vaporization of water, and the second step at 220–380 °C was the relatively rapid decomposition. Secondary *y*-axis shows derivative thermogravimetry of cellulose nanocrystals from 25 °C to 600 °C.

The temperature stability of CLCN is a crucial characteristic, particularly in applications that engage high temperatures. To assess this, a thermogravimetric study on CLCN was conducted, and the thermal decomposition along with the first derivative, is illustrated in Figure 2. The weight loss of CLCN commenced at 35 °C, with a loss of 7.56% of mass observed by the time the temperature reached 100 °C. This initial weight loss is due to the evaporation of water present in CLCN (Huang et al., 2014). Additionally, thermogravimetric analysis indicated that as the temperature reached 380 °C, more than 89% of the initial mass was lost. Upon reaching 500 °C, a substantial 95% loss of the initial mass of CLCN was recorded. This finding aligns with the thermogravimetric characteristics of the thermal stability of cellulose-chitosan aerogels (Rostamitabar et al., 2022).

#### 3.2.3 Differential scanning colorimetry (DSC)

**Figure 3:**
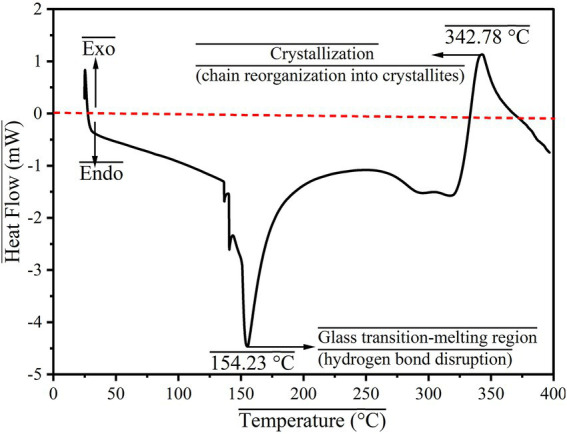
DSC curve for CLCN.

In order to verify the degradation of the synthesized aerogel material with respect to heat flow, DSC analysis was performed in a temperature range of 10 to 400 °C. The results are presented in Figure 3, in which the material shows two endothermic peaks and one exothermic peak. The first endothermic peak appearing at 60.45 °C is due to the removal of adsorbed humidity from the nanoparticle’s surface. The second endothermic peak appeared at 131.95 °C, is due to the melting or fusion of the material. At this stage, the polysaccharide undergoes 1,4-linked bond breakage, pyranose chair distortion under temperature variation. The exothermic peak appearing at 250.63 °C is due to the crystallization process of the fused material. The DSC curve of the aerogel supports the TGA data.

#### 3.2.4 Scanning electron microscopic (SEM) analysis

**Figure 4:**
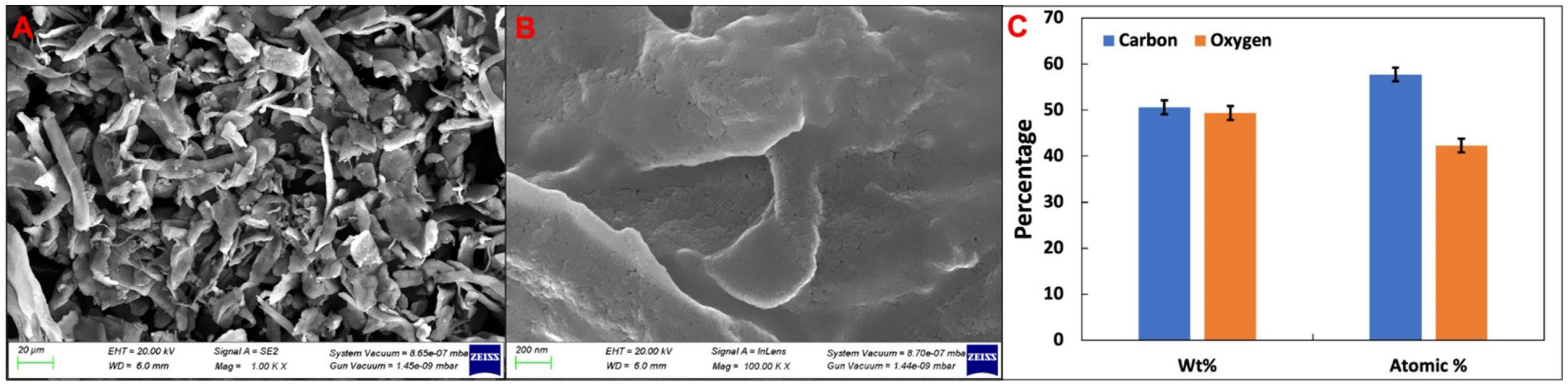
**(A)** SEM image of CLCN at 1000× magnification. **(B)** SEM image of CLCN at 100000X magnification. **(C)** Elemental analysis of cellulose nanocrystals.

The surface morphology of the CLCN was examined through SEM. Figures 4A,B displays SEM images of CLCN at both 1,000× and 100,000× magnifications. The images revealed CLCN with wrinkled surfaces and distinctive three-dimensional grooves (Dharunya et al., 2016). The grooves exhibited a uniform and compact pattern with reduced pore distribution, possibly attributed to increased cross-linking. This heightened cross-linking enhances both intra- and inter-molecular interactions, contributing to the formation of well-interconnected three-dimensional CLCN (Dharunya et al., 2016). It’s noteworthy that surface characteristics of nanomaterials play a pivotal role that controls cell attachment (Boyan, 1996). The three-dimensional surface serves as the primary interacting site, facilitating cell growth in layers and entanglement within the matrix. The results indicate that CLCN, characterized by grooved porous 3D matrices, can serve as effective scaffolds, accommodating numerous cells on the grooved surfaces (Tabata et al., 1994). These findings align with a previous report (Revin et al., 2020). The elemental composition of the analysis was done using EDX, and the data are presented in Figure 4C. The data revealed the presence of carbon and oxygen as 50.628 and 49.372% by weight, respectively. In terms of atomic percentage, the relative amount of carbon and oxygen was 57.73 and 42.27%, respectively. Furthermore, it is well-established that the concentration of sulfuric acid during hydrolysis strongly influences CNC morphology, crystallinity, and surface sulfate groups, thereby affecting colloidal stability and particle size distribution (Arserim-Uçar et al., 2021).

### 3.3 Determination of MIC

MIC of the synthesized CLCN against the *P. aeruginosa* PAO1, *E. coli* ATCC 25922, *C. violaceum* ATCC 12472, and *S. marcescens* MTCC 97 was observed as 2 mg/mL, while against *L. monocytogenes* ATCC 19114 it was recorded to be 8 mg/mL. On the other hand, MIC of curcumin against test bacterial strains ranged from 512–1,024 μg/mL. The highest MIC (1,024 μg/mL) of curcumin was observed in the case of *C. violaceum* ATCC 12472, *E. coli* and *S. marcescens* MTCC 97. In *L. monocytogenes* ATCC 19114, the recorded MIC was 512 μg/mL. All assays were conducted using concentrations below the MIC, referred to as sub-MICs.

### 3.4 Inhibition of violacein production by CLCN

**Figure 5:**
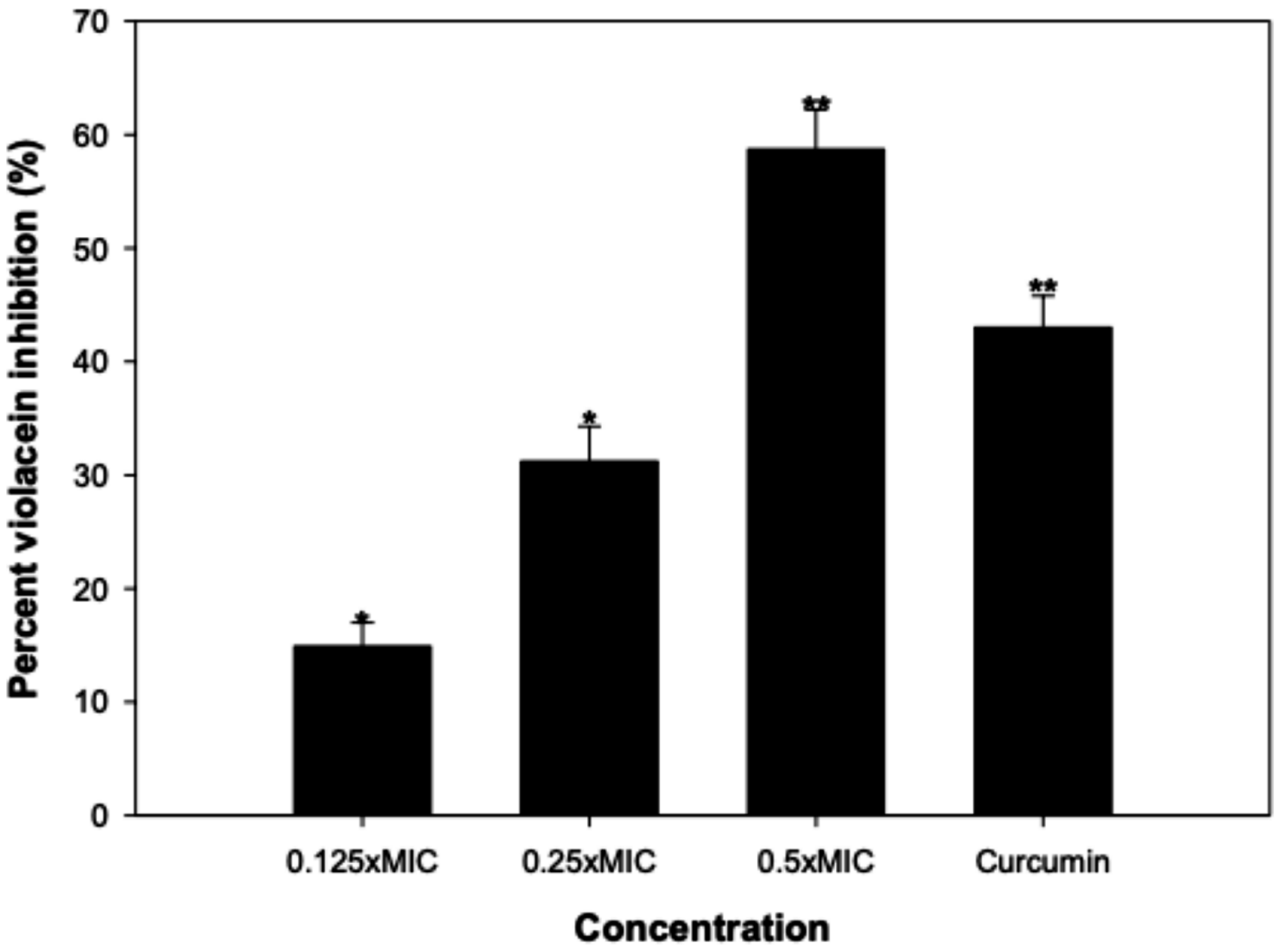
Effect of sub-MICs of CLCN and curcumin on violacein production in *C. violaceum.* Percent inhibition was calculated relative to the untreated control. Values are mean ± SD (*n* = 3). Statistical comparisons were performed using one-way ANOVA. *p* < 0.05 (*), *p* < 0.01 (**).

The spectrophotometric assessment of violacein was conducted quantitatively. The data revealed that the absorbance of *C. violaceum* 12,472 treated with CLCN consistently decreased with increasing concentrations, as depicted in Figure 5. In the presence of 0.25 and 0.5 mg/mL of CLCN, there were reductions in pigment production by 14.9 and 31.2%, respectively. The highest sub-MIC tested (1 mg/mL) resulted in a significant 59% inhibition of pigment production. Treatment with free curcumin at the highest test sub-MIC (512 μg/mL) resulted low level of inhibition (43%) compared to CLCN. These results affirm the inhibitory impact of CLCN on QS-regulated violacein pigment production in *C. violaceum* 12,472. In short, CLCN demonstrated a sufficient level of violacein inhibition. The findings align with a prior study where tea polyphenols extracted successfully inhibited violacein pigment by more than 80% (Yin et al., 2015). In another study, ZnO-curcumin nanocomposites exhibited reduced violacein production in *C. violaceum* CVO26 due to reduced binding of homoserine lactone (signal molecule) with its receptor (Prateeksha et al., 2019).

### 3.5 Inhibition of prodigiosin by CLCN

**Figure 6:**
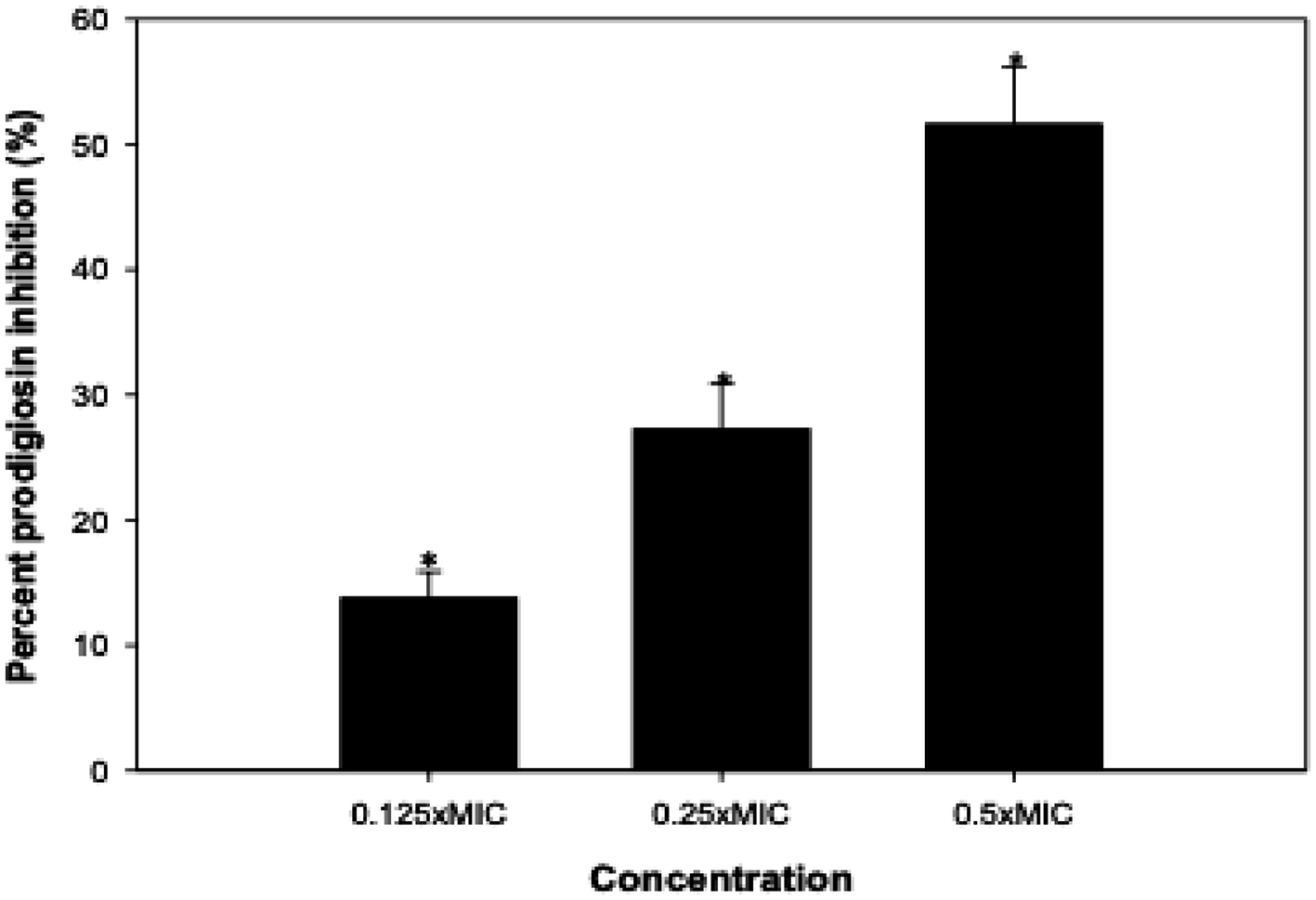
Effect of sub-MICs of CLCN on prodigiosin production in *S. marcescens.* Percent inhibition was calculated relative to the untreated control. Values are mean ± SD (*n* = 3). Statistical comparisons were performed using one-way ANOVA. *p* < 0.05 (*), *p* < 0.01 (**).

Prodigiosin constitutes a red pigment in *S. marcescens*, and its synthesis is governed by quorum sensing (Morohoshi et al., 2007). The data presented in Figure 6 reveal the suppression of this pigment production by CLCN. The inhibition percentages for the pigment were measured at 13.8, 27.3, and 51.6% when treated with 0.25, 0.5, and 1 mg/mL of CLCN, respectively. Some strains of *S. marcescens* indicate the existence of shared regulatory mechanisms linking prodigiosin biogenesis to other phenotypes such as flagellar variation, protease production, and hemagglutination (Goluszko et al., 1995). In a recent study, a nano-formulation of halloysite nanotubes loaded with curcumin was reported to impair the synthesis of prodigiosin in *S. marcescens* significantly (Fakhrullina et al., 2019).

### 3.6 CLCN inhibit the QS-controlled virulence factors of *P. aeruginosa* PAO1

**Figure 7:**
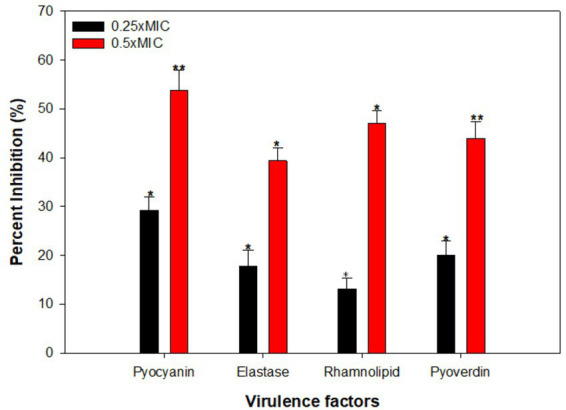
Effect of sub-MICs of CLCN on QS-regulated virulence factor production in *P. aeruginosa* PAO1. Percent inhibition was calculated relative to the untreated control. Values are mean ± SD (*n* = 3). Statistical comparisons were performed using one-way ANOVA. *p* < 0.05 (*), *p* < 0.01 (**).

Pyocyanin, characterized by its blue-greenish pigment, serves as one of the known virulence factors in *P. aeruginosa* (Gupta et al., 2017). Treatment with CLCN demonstrated inhibition of pyocyanin levels, as outlined in Figure 7. At 0.5 and 1 mg/mL, there was significant inhibition of 29 and 53% in pyocyanin production, respectively. Pyocyanin is well-recognized to take part in the pathogenicity of *P. aeruginosa* and interfere with various cellular functions (Ran et al., 2003). Phenazine-1-carboxylic acid (a pyocyanin precursor) and the pyocyanin disrupt the proper beating of respiratory cilia in humans and also perturb the expression of immunomodulatory proteins in individuals with cystic fibrosis (Fothergill et al., 2007). Furthermore, pyocyanin also suppresses the host’s defence system, causing an increased apoptosis rate in human neutrophils, in addition to its supportive role in biofilm development (Das et al., 2015). Pyocyanin also induces oxidative stress, which is found to be positively correlated with the severity of the disease as well (Hunter et al., 2012). Similarly, in a study conducted to evaluate the anti-virulence potential of curcumin-metal complexes against *P. aeruginosa* PAO1, it was reported that sub-MICs of metal (Cu, Fe, and Zn)-curcumin complexes reduced pyocyanin production significantly. The highest inhibition of 76.5% in pyocyanin production was recorded with Cu-curcumin complex at ¼ MIC (Gholami et al., 2020). Further, our results on reduced pyocyanin production are in agreement with those reported with meta-bromo-thiolactone-loaded calcium alginate nanoparticles (CANP). Significant inhibition of pyocyanin pigment was found in *P. aeruginosa* treated with 0.5 mg/mL concentration of these nanoparticles (Eltayb et al., 2022).

Pyoverdin is another virulence factor of *P. aeruginosa*. This siderophore plays a crucial role in virulence and the infection (Peek et al., 2012). The addition of 0.5 and 1 mg/mL of CLCN decreased the pyoverdin levels by 20 and 44%, respectively, with respect to the control (Figure 7). The solvent control does not exhibit any significant effect on the siderophore levels. Pyoverdin plays a role in pathogenesis by sequestering transferrin protein in mammals, leading to a deficiency of iron in the host tissues (Das et al., 2016). This siderophore successfully avoids detection by lipocalin, which is linked to neutrophil gelatinase, thereby facilitating the onset of *P. aeruginosa* infections, particularly in the lungs of cystic fibrosis patients (Peek et al., 2012). Consequently, it is expected that any chemotherapeutic agent that decreases pyoverdin production would reduce the virulence of these bacteria.

Our results are further strengthened with the findings on reduced pyoverdine production in *P. aeruginosa* PAO1 at all tested concentrations of multi-walled carbon nanotubes (MWCNT) (Jabłońska et al., 2022). In another study, titanium-cerium nanocomposite at a 400 μg/mL concentration decreased the pyoverdine production by 53.8% (Altaf et al., 2023).

The ability of CLCN to reduce elastase activity, which is dependent on quorum sensing and degrades elastin, was evaluated. The results reveal a reduction of elastases in the supernatant of *P. aeruginosa* PAO1 when compared to the control (Figure 7). CLCN demonstrated a reduction in LasB elastase activity by 17 and 39% upon treatment with 0.5 and 1 mg/mL concentration. *Pseudomonas aeruginosa* secretes a range of hydrolytic enzymes, such as elastases, which play a crucial role in breaking down tissue components and disrupting the host’s immune defense (Bandara et al., 2006). Previous studies have shown that sub-minimal inhibitory concentrations (sub-MICs) of 6-Gingerol can reduce the expression of *lasB* gene, which is responsible for coding elastase (Bandara et al., 2006). Concentration-dependent inhibition of elastase was recorded with green-synthesized chitosan-selenium bionanocomposite (CS-SeNC) in PAO1. Reduced elastase in CS-SeNC-treated PAO1 was attributed to the decreased expression of *lasB* gene of the LasIR QS systems (Karthick Raja Namasivayam et al., 2022). Since, expression of Las proteins plays a crucial role in QS-governed virulent phenotypes and biofilm development. The reduction in elastolytic enzymes indicates the potential inhibition of the LasIR QS system by CLCN.

The rhamnolipid production in *P. aeruginosa* PAO1 was also inhibited when treated with CLCN (Figure 7). At 0.5 and 1 mg/mL, there was a reduction in rhamnolipid production by 13 and 47%, respectively. Rhamnolipids are crucial for QS-controlled swimming motility, and they also assist in biofilm dispersal at sites of infection (Köhler et al., 2000). Acting as a surfactant, rhamnolipids facilitate surface movement and are essential for the initial stages of biofilm formation (O’May and Tufenkji, 2011). Therefore, it is envisaged that any interference with the synthesis of rhamnolipid in PAO1 is bound to affect biofilm biofilm-forming capabilities of the pathogen. A similar investigation was reported earlier, where treatment with plumbagin reduced the rhamnolipid production in *P. aeruginosa* PAO1 by 49% (Qais et al., 2021). Furthermore, our finding gets support from a recently published investigation, wherein bio-fabricated AgNPs significantly reduced rhamnolipid production in PAO1 at concentrations ranging from 7.81–31.25 μM (Saeki et al., 2022).

### 3.7 CLCN mitigate the total exoproteases

**Figure 8:**
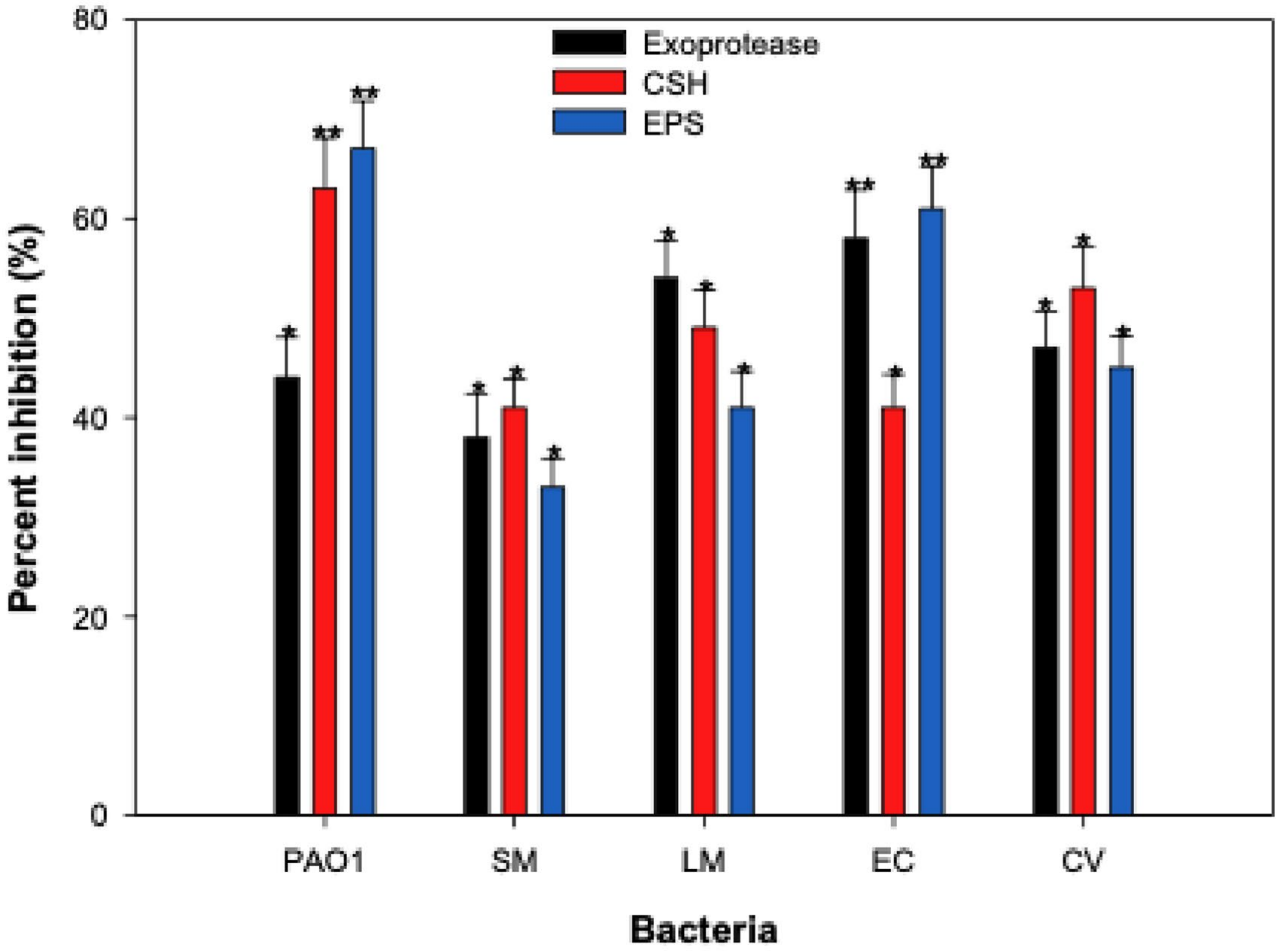
Effect of sub-MICs of CLCN on virulence factors associated with biofilm formation in test bacteria. Percent inhibition was calculated relative to the untreated control. Values are mean ± SD (*n* = 3). Statistical comparisons were performed using one-way ANOVA. *p* < 0.05 (*), *p* < 0.01 (**).

The comparative quantification of total exoproteases in culture supernatant without and with CLCN was performed using the azocasein-degradation protocol. Treatment with 1 mg/mL of CLCN exhibited inhibition of protease production in *P. aeruginosa* PAO1 by 44%, compared to the control (Figure 8). Similarly, the total proteases in *S. marcescens*, *E. coli* and *C. violaceum* were reduced by 38, 58 and 47%, respectively, at 1 mg/mL CLCN. Inhibition of 54% in exoprotease production was recorded in *L. monocytogenes* ATCC 19114 at 0.5xMICs (4 mg/mL) of CLCN. Proteases enhance bacterial invasion by breaking down host cell proteins and bypassing the host’s immune defense (Smith and Iglewski, 2003).

### 3.8 Inhibition of exopolysaccharides production by CLCN

Extracellular polymeric substances are high molecular weight natural polymers that form the structural backbone of biofilms (Maunders and Welch, 2017). These substances are essential for biofilm formation and significantly influence their physicochemical characteristics. Among its various components, exopolysaccharides (EPS) are the primary contributors (Sauer and Camper, 2001). EPS create a protective shield around bacterial cells, preventing the penetration of chemotherapeutic agents like antibiotics, thereby acting as a defense mechanism against antimicrobial treatments. Increased production of EPS modifies the biofilm structure and enhances drug resistance (Sauer and Camper, 2001). There is a strong correlation between biofilm formation and EPS secretion, suggesting that inhibiting EPS production could be a viable strategy for disrupting biofilms (Czaczyk and Myszka, 2007). CLCN was found to inhibit the EPS levels in test bacteria (Figure 8). At 0.5xMICs, EPS secretion by *P. aeruginosa*, *S. marcescens*, *E. coli* and *C. violaceum* decreased by 67, 33, 61 and 45%, respectively. Similarly, the presence of 4 mg/mL of CLCN reduced the EPS production in *L. monocytogenes* ATCC 19114 by 41%.

EPS is an important part of the architecture of biofilms, providing structural stability and protecting bacteria from environmental stresses and antibiotics (Flemming et al., 2007). Consequently, focusing on EPS reduction is viewed as an alternative approach to hinder biofilm formation, as diminishing EPS secretion is anticipated to negatively impact the biofilm-forming capabilities of bacteria. Similarly, Significant EPS reduction in MRSA, *L. monocytogenes*, *S. marcescens*, *E. coli*, and *P. aeruginosa* was found upon treatment with sub-MICs gold-graphene oxide-based nanocomposites (Aljaafari et al., 2020).

### 3.9 Measurement of cell surface hydrophobicity (CSH)

The impact of CLCN on CSH was assessed, given that CSH plays a vital role in the adherence of bacteria to solid surfaces and the formation of biofilms (Salini and Pandian, 2015). CLCN exhibited a significant reduction in the CSH of test pathogenic bacteria at 0.5xMICs. At 1 mg/mL of CLCN, the CSH of *P. aeruginosa*, *S. marcescens*, *E. coli*, and *C. violaceum* decreased by 63, 41, 43, and 53% respectively, as illustrated in Figure 8. Similarly, the CSH of *L. monocytogenes* decreased by 49% when treated with 0.5xMIC (4 mg/mL) of CCA. This finding aligns with previous research demonstrating reduced hydrophobicity of *E. coli* and *S. aureus,* leading to decreased adhesion of cells upon treatment with polydimethyl siloxane nanocomposites (Sankar et al., 2017). Since CSH has a significant role in the adhesion of bacterial cells to the surface for biofilm formation, our findings on reduced CSH in the test bacteria is of utmost importance.

### 3.10 Inhibition of biofilm development by CLCN

**Figure 9:**
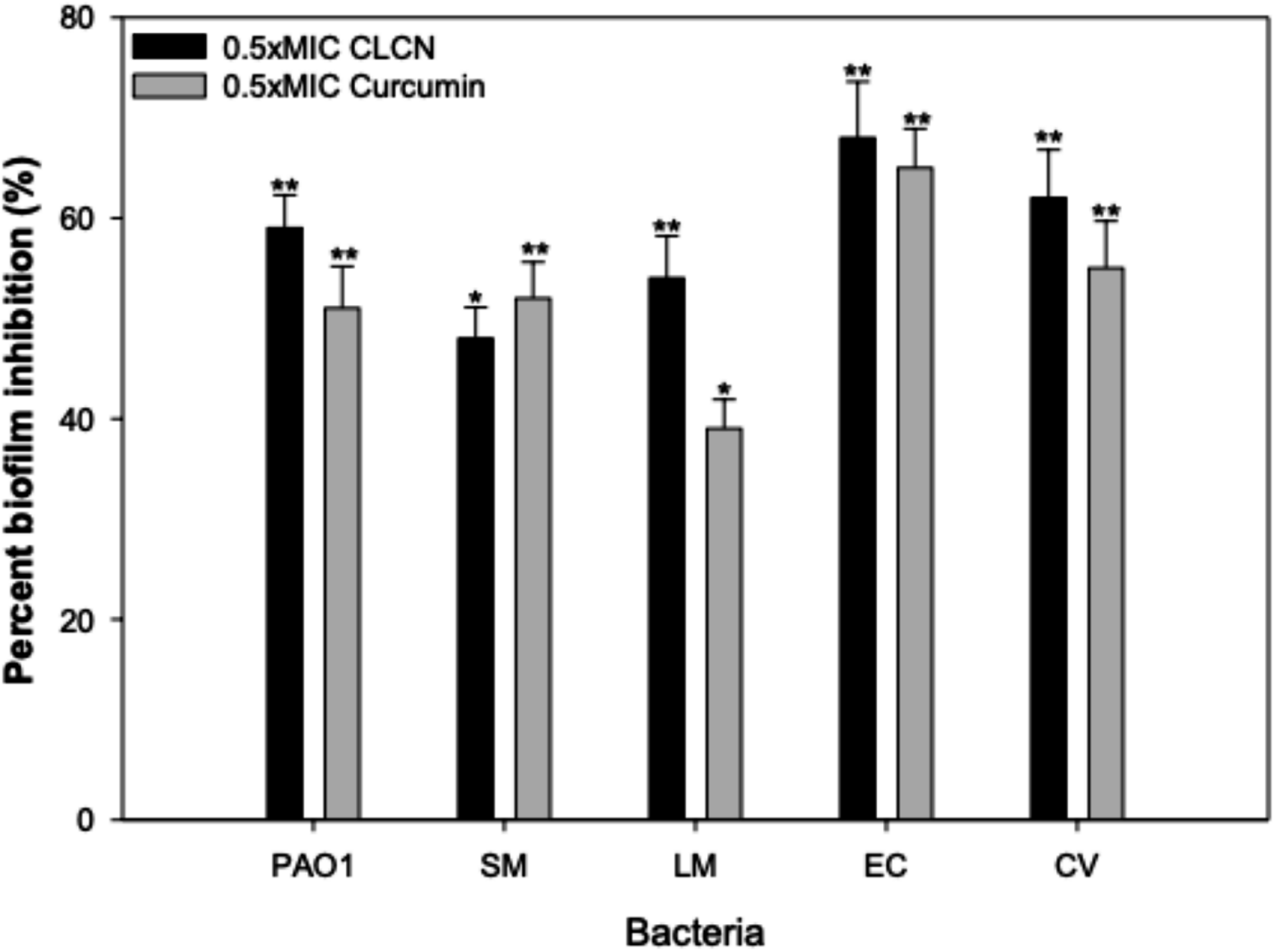
Effect of sub-MICs of CLCN and curcumin on biofilm formation in test bacteria. Percent inhibition was calculated relative to untreated biofilms. Values are mean ± SD (*n* = 3). Statistical comparisons were performed using one-way ANOVA. *p* < 0.05 (*), *p* < 0.01 (**).

**Figure 10:**
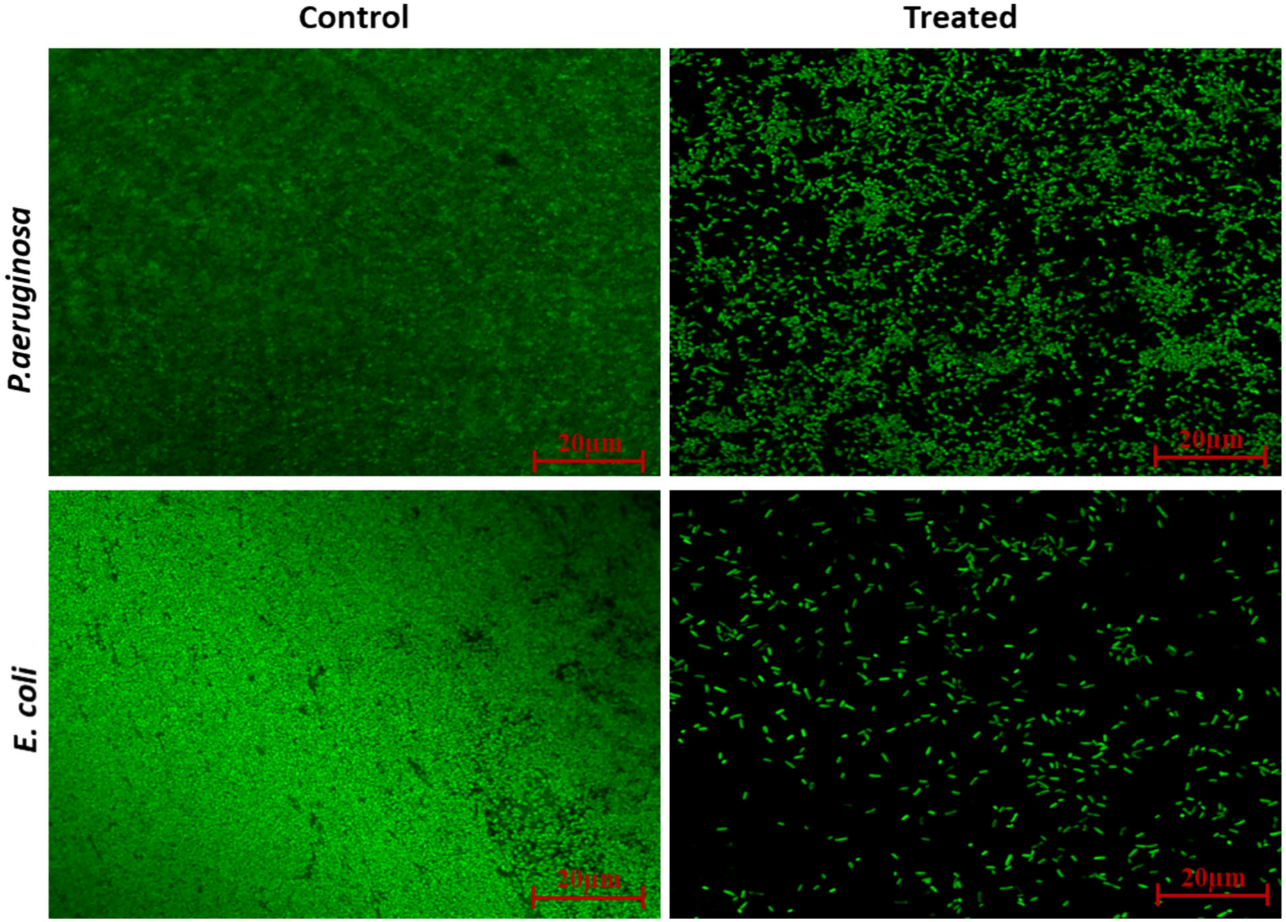
CLSM images of CLCN untreated (control) and treated biofilms of *P. aeruginosa* and *E. coli* at the highest test sub-MICs (0.5xMICs).

As shown in Figure 9, CLCN reduced biofilm development remarkably in all test pathogens at 0.5xMIC. Treatment with 1 mg/mL of CLCN resulted in reduced biofilm in *P. aeruginosa*, *S. marcescens*, *E. coli*, and *C. violaceum* by 59, 48, 68, and 62%, respectively, when compared to the control. The treatment of 4 mg/mL of CLCN reduced the biofilms of *L. monocytogenes* by 54%. In comparison, curcumin at 0.5 MIC (512 μg/mL) exhibited inhibitory effects of 51, 52, 39, 65, and 54% against *P. aeruginosa*, *S. marcescens*, *E. coli*, and *C. violaceum*, respectively. The quantitative data on biofilm inhibition were validated using CLSM. Untreated cultures of *Pseudomonas aeruginosa* PAO1 and *Escherichia coli* ATCC 25922 developed dense, mat-like biofilms on glass coverslips, as depicted in Figure 10. However, the introduction of CLCN significantly diminished the biofilm-forming capacity of the pathogens, leading to a loss of their clustered structure. Most of the treated cells remained in a planktonic state. Additionally, CLSM images of the treated cells confirmed that CLCN reduces microbial adherence and subsequent biofilm formation (Figure 10). The micrographs showed a scattered distribution of CLCN-treated cells, with fewer instances of thick biofilm clusters due to decreased cohesiveness compared to the control.

The ability of *Pseudomonas aeruginosa* to cause infections is largely due to its biofilms, which enhance its resistance to both physical and chemical treatments. The development of these biofilms is a highly regulated and organized process, closely linked to bacterial quorum sensing (Qin et al., 2015). Research indicates that biofilms play a role in about 80% of human infections. The biofilm is a complex matrix made up of extracellular polymeric substances EPS, lipids, and proteins (Flemming and Wingender, 2010). Earlier, we have demonstrated statistically significant reduction in biofilms of bacterial pathogens upon application of sub-MICs of dextrin-based poly (methyl methacrylate) grafted silver nanocomposites and gold-graphene oxide-based nanocomposites (Aljaafari et al., 2020; Hasan et al., 2019). Further, in a recent study, curcumin-loaded nanofibers were observed to reduce biofilm formation in PAO1 and *S. mutans* (Di Salle et al., 2021).

Taken together, the enhanced inhibitory effects observed for CLCN compared to free curcumin across both violacein production and biofilm formation clearly demonstrate the functional advantage of CLCN. Cellulose-loaded nanoparticle formulation enhances curcumin’s stability, bioavailability, cellular uptake and interaction with bacterial signalling systems. Thus, it is envisaged that the synthesised materials could prove effective in mitigating bacterial biofilm and preventing biofilm-based persistent infections.

### 3.11 Disruption of preformed biofilms by CLCN

**Figure 11:**
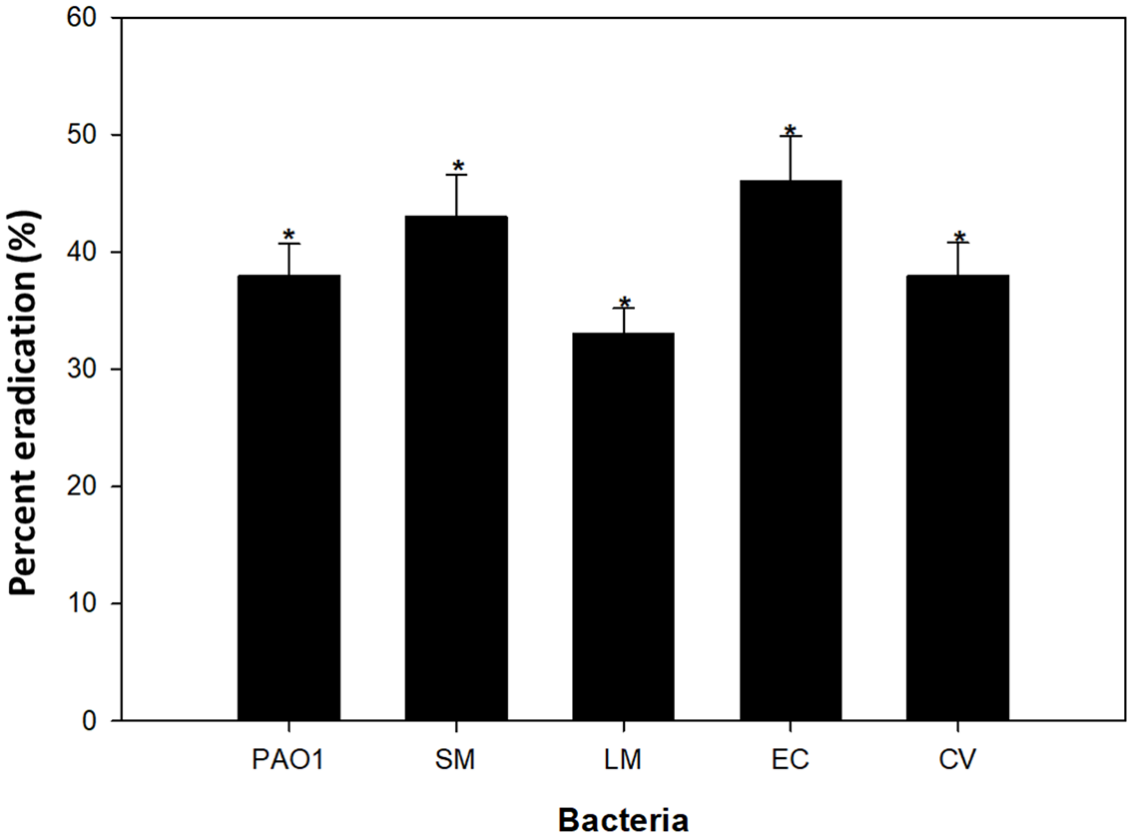
Effect of sub-MICs of CLCN on pre-formed (mature) biofilms in test bacteria. Percent eradication was calculated relative to untreated mature biofilms. Values are mean ± SD (*n* = 3). Statistical comparisons were performed using one-way ANOVA. *p* < 0.05 (*), *p* < 0.01 (**).

Majority of antibiotics target the planktonic form of bacterial growth, with only a limited number effective against biofilm formation (Roy et al., 2018). Preventing biofilm formation is generally simpler than eliminating already established biofilms. In many infections, biofilms successfully establish themselves at the infection site. In this study, bacterial strains were first allowed to form biofilms, which were then treated. The impact of CLCN on the removal of these preformed biofilms is illustrated in Figure 11. In the presence of 0.5xMIC of CLCN eradicated the established biofilms of *P. aeruginosa*, *S. marcescens*, *L. monocytogenes*, *E. coli*, and *C. violaceum* by 38, 43, 33, 46 and 38%, respectively.

The results indicate that the test bacteria’s biofilms were effectively eradicated. Biofilms consist of clusters of bacterial cells encased in extracellular polymeric substances (Li et al., 2020). Their primary constituents include polysaccharides, nucleic acids, and polypeptides, along with various other biochemical elements. These components form a barrier that impedes the penetration of antibiotics to bacterial cells (Høiby et al., 2011). Thus, the removal of mature biofilm is tough. Here, we report broad-spectrum removal of established biofilms of *E. coli*, *P. aeruginosa*, *S. marcescens*, *L. monocytogenes*, and *C. violaceum*. The data align with earlier research that demonstrated the successful disruption of mature biofilms formed of *P. aeruginosa*, *E. coli*, MRSA, and *L. monocytogenes* (Al-Shabib et al., 2020). Furthermore, our results are also consistent with studies using curcumin-loaded starch aerogels, which also demonstrated inhibition of QS-regulated virulence and biofilm formation, indicating that biopolymer carriers can enhance curcumin’s biological efficacy (Mabood Husain et al., 2024). The results clearly show that CLCN were successful both in the inhibition and eradication of the biofilms of the test bacteria.

## 4 Conclusion

The dramatic rise in antimicrobial resistance over the past two decades has underscored the need for innovative solutions in combating infectious diseases. In this study, curcumin-loaded cellulose nanoparticles (CLCN) exhibited broad-spectrum antibiofilm, anti-quorum-sensing, and anti-virulence activities against major food-borne pathogens, including *P. aeruginosa*, *E. coli*, *S. marcescens*, *C. violaceum*, and *L. monocytogenes*. To the best of our knowledge, this is the first report demonstrating the broad-spectrum anti-QS and antibiofilm efficacy of CLCN, highlighting its potential as a natural, biocompatible anti-infective agent. These findings suggest that CLCN can be further developed for food safety applications, such as antimicrobial packaging and surface coatings, to limit contamination by drug-resistant bacteria. Future studies will also assess solvent residues, loading efficiency, release and safety profile of curcumin to support practical application. Additionally, the versatile properties of CLCN make it a promising candidate for biomedical applications, treatment of persistent infections, tissue engineering and wound dressing, ultimately contributing to improved public health outcomes and the sustainability of current antimicrobial strategies.

## Data Availability

The raw data supporting the conclusions of this article will be made available by the authors, without undue reservation.
